# Association between rotavirus gastroenteritis and intussusception: suggested evidence from a retrospective study in claims databases in the United States

**DOI:** 10.1080/21645515.2020.1770514

**Published:** 2020-07-01

**Authors:** Corinne Willame, Brigitte Cheuvart, Emmanuel Aris, Volker Vetter, Catherine Cohet

**Affiliations:** GSK Vaccines, Wavre, Belgium

**Keywords:** Rotavirus gastroenteritis, intussusception, self-controlled case series, claims databases

## Abstract

The etiology of intussusception (IS), a serious gastrointestinal obstruction, remains unclear. Limited evidence suggests a role for viral infection. We investigated the risk of IS after rotavirus gastroenteritis (RV GE) in the first year of life. In this retrospective, self-controlled case series (SCCS), we assessed the risk of IS after RV GE using data from United States administrative claims databases. Incidence rate ratios (IRR) of IS were calculated for the 7- and 21-day risk periods after RV GE (main analysis) or after fracture (sensitivity analysis). A total of 290,912,068 subjects were screened; 42 presented claims for RV GE and IS, and 66 for fracture and IS. The IRRs of IS after RV GE were 79.6 (95% confidence interval, CI: 38.6–164.4) and 25.5 (95% CI: 13.2–49.2) in the 7- and 21-day risk periods. The sensitivity analysis showed an association between IS and fracture for both periods, suggesting potential confounding. Post-hoc analyses did not confirm the association between fracture and IS but suggested a potential association between RV GE and IS. A temporal association between RV GE and IS was detected using claims databases. Due to some limitations of the data sources, this association should be further investigated.

## Introduction

Intussusception (IS) is a serious, potentially life-threatening medical condition of acute intestinal obstruction. It occurs when a segment of the bowel prolapses into a more distal portion, which can cause intestinal ischemia, infarction and perforation.^1^

Between 60% and 70% of children diagnosed with IS are <1 year of age, with most episodes occurring between 5 and 7 months of age.^2^ IS affects mostly healthy infants and presents a male to female ratio of 3:2.^3^ Estimates of IS incidence vary broadly across geographical regions, with the highest rates observed in Asia.^2^ Incidence rates in Europe and United States (US) are consistently similar, ranging from 20 to 66 cases per 100,000 person-years.^2,4,5^

To date, the etiology of IS remains unclear. Studies assessing potential risk factors for pediatric IS point toward lipomas, neuronal intestinal dysplasia, Celiac or Chron’s diseases, cystic fibrosis, malignancies, gastroenteritis, and parasitic or viral infections.^6,7^ Following common viral illnesses such as those caused by adenoviruses or rotaviruses, IS may result from hypertrophy of the Peyer patches.^6^ While an association with adenovirus infection has been demonstrated,^8,9^ evidence of an association with rotavirus (RV) infection is still limited.^9–11^

The implementation of RV vaccination in national immunization programs across the world has resulted in effective and substantial reductions in mortality and morbidity associated with RV GE (rotavirus gastroenteritis).^12^ However, the use of live-attenuated RV vaccines at a large scale demonstrated an increased risk of IS during the first week after immunization: a meta-analysis estimated the relative risk of IS after the first dose of both live-attenuated RV vaccines as 5.4 and 5.5, and the risk after the second dose as 1.7 and 1.8.^13^ Because the currently used RV vaccines are live-attenuated and replicate in the gut, the observed association between RV vaccination and IS supports the potential role of natural RV infection in the development of IS. However, to date, there is no robust evidence available on the risk of IS following RV GE.

The self-controlled case series (SCCS) method has been developed for the specific purpose of detecting acute adverse events following immunization.^14,15^ Each case serves as its own control, as the observation period is divided into control and risk periods (i.e. pre-defined post-exposure period). The outcome measure is the incidence rate ratio (IRR) between the risk and control periods.^16^ The SCCS allows to control implicitly for confounders that do not vary with time (such as genetics, underlying conditions, socio-economic status) and for age and temporal variations (such as seasonality, infections) in the baseline incidence. It has previously been applied, for example, in observational studies to show the association between vaccine-preventable infectious diseases such as varicella, influenza, or pneumococcal pneumonia, and cardiovascular disease.^17–19^ The known association between live-attenuated RV vaccines and IS suggests that RV infection may play a role in the development of IS. The SCCS has been used extensively to assess the association between RV vaccination and IS,^13,20,21^ and was therefore considered fully appropriate for the present investigation of the association between RV GE and IS.^14,22^

In this retrospective study, we explored the association between RV GE and IS in children <1 year of age using data extracted from administrative claims databases in the United States of America (US). The aim of the study was to measure and compare the incidence of IS between pre-defined risk and control periods by applying the SCCS study design. The risk of IS was assessed for periods of 7 and 21 days following an RV GE episode.

**Figure 1.**
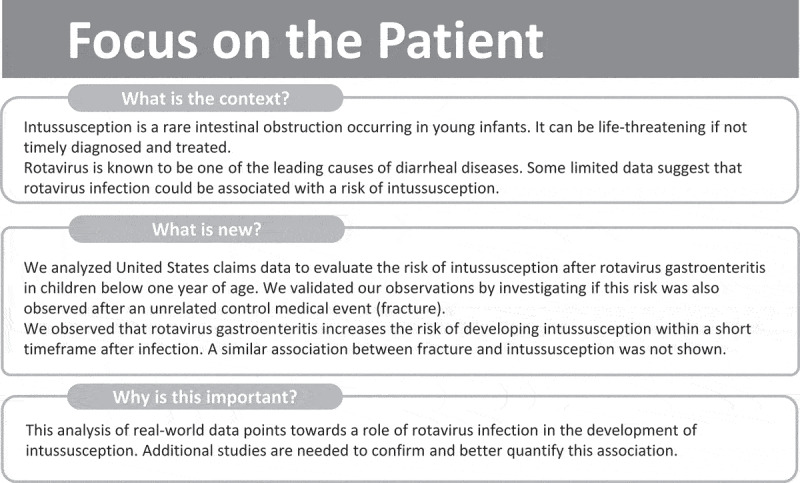
Focus on the Patient Section

A summary contextualizing the outcomes of this study is displayed in the Focus on the Patient Section (Figure 1) for the information of health-care professionals.

## Methods

### Data sources and study population

This retrospective, observational, SCCS analyzed data from two US insurance claims databases: Truven Health MarketScan (“MarketScan,” consisting of Commercial Claims and Encounter databases and Medicaid databases) and Optum Clinformatics Data Mart (“Optum”). These administrative databases contain individual-level anonymized data such as socio-demographic information, clinical diagnosis, drug prescription, and procedures for in- and outpatients across the US who are covered by fee-for-services and managed care plans.^15,23^ MarketScan contains data of >240 million unique patients since 1995, whereas Optum contains data of >13 million unique patients annually since 2000.

The study included individual-level data in the following periods during which GSK had a license to access the data: January 2003 to December 2017 for MarketScan, and from May 2000 to December 2017 for Optum.

The primary objective was to assess the risk of IS during the 7-day period following RV GE in subjects <1 year of age. The secondary objective was to assess the risk of IS during the 21-day period following RV GE in the same population. These risk periods are now universally accepted in studies of the risk of RV vaccination using the SCCS design.^13^ Sensitivity analyses were conducted for fracture, a control medical event for which no plausible biological relationship with IS exists, hence used as an indicator of the quality of the data sources.

### Subjects, exposure, and outcomes

Subjects <1 year of age who remained in a health-care plan until the age of 1 year and for whom both RV GE or fracture claims, and claims for IS, were reported while on this plan between May 2000 and December 2017 were included.

Eligible subjects were classified into two groups: (1) RV GE group: subjects <1 year of age with an International Classification of Diseases (ICD) code for RV GE (ICD-10: A08.0; ICD-9: 008.61) and an ICD code for IS (ICD-10: K56.1; ICD-9: 560.0); and (2) fracture group: subjects <1 year of age with an ICD code for fracture except for clavicle fracture and fracture at birth (code list available upon request) and an ICD code for IS.

All subjects with an RV vaccination claim on the same day as an RV GE claim were excluded from the RV GE group, since it is unlikely that a child with gastroenteritis symptoms receives an RV vaccine, or that RV vaccination would trigger RV GE on the same day. RV vaccination claims were identified through current procedural terminology (CPT) codes 90680 or 90681 or national drug codes (NDC) 00006404701, 00006404720, 00006404731, 00006404741, 00008256201, 58160080501, 58160080502, 58160080511, 58160085101, 58160085110, 58160085302, or 58160085452.

Claims for clavicle fracture and fracture at birth (i.e. fractures with a description containing the term “birth,” “clavicle,” or “clavicule”) were excluded from the fracture group since they are frequently reported in newborns.

Subjects were first screened by identifying RV GE, fracture, and IS claims in both databases. The Commercial and Medicaid databases belonging to MarketScan were screened separately but data were pooled. Since the databases only provide the year of birth, the complete date of birth (i.e. day, month, and year) was defined as the date of earliest birth diagnosis code recorded, if available, the date of the earliest record of medical diagnosis under insurance coverage, or the first date of insurance coverage. If the imputed date of birth did not coincide with the year of birth recorded in the databases, the subject was excluded from the analysis. Among screened subjects, those with RV GE or fracture and IS claims in their first year of life were selected. Since one episode of IS, RV GE, or fracture could be associated with more than one claim, we considered that all recurring claims for a particular episode identified in a period <30 days corresponded to a single episode.

For each eligible subject identified, the following data were extracted from the database: year of birth, gender, date of claims (IS, RVGE, and/or fracture), and date of RV vaccination (if available).

This study was designed and conducted according to the principles of the Declaration of Helsinki, Good Epidemiological Practices, and the International Conference on Harmonization (ICH) guidelines for clinical investigation of medicinal products in the pediatric population (ICH E11). Data from Truven Health MarketScan Commercial Claims and Encounters and from Optum Clinformatics Data Mart are anonymized and comply with the Final Privacy Rule of the Health Insurance Portability and Accountability Act (HIPAA) of 1996; therefore, no subject-informed consent is required for studies using these data sources.

### Statistical methods

For each group, the demographic characteristics, history of RV vaccination, age at IS, age at RV GE or fracture, and number of IS episodes (during both the risk and control periods) were summarized using descriptive statistics. We also calculated the total subject-weeks during the risk and control periods and the number of days between RV GE or fracture and IS (calculated as the date of IS minus date of RV GE or fracture plus 1 day).

All statistical tests were two-sided with a significance level of alpha = 0.05. Statistical analyses were performed using Statistical Analysis Software (SAS) version 9.3.

#### Main analysis

**Figure 2.**
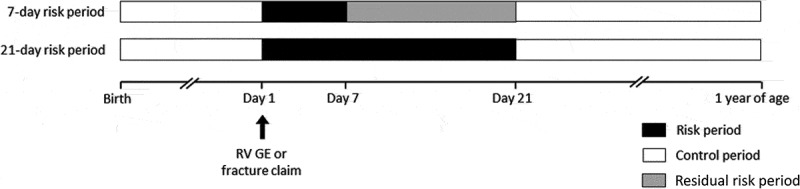
Study design. RV GE, rotavirus gastroenteritis. Day 1 was defined as the day of RV GE or fracture claim. The residual risk period was not taken into consideration in the analyses

For the primary and secondary objectives of the study, we applied the SCCS method to data from the RV GE group (Figure 2). We considered the day of RV GE claim as Day 1. The risk period was defined as the period from Day 1 to Day 7 for the primary objective, and from Day 1 to Day 21 for the secondary objective. The control period was defined as the period from birth until 1 year of age excluding the period from Day 1 to Day 21 after vaccination. Due to a potential residual risk of IS reporting between Day 8 and Day 21 (also called buffer period in the SCCS method), this interval was excluded from the control period for the analysis of the primary objective (Figure 2). Consequently, the same control periods were used for both objectives.

The null hypothesis was that the incidence rate of IS during the risk period is the same as that during the control period. To test this hypothesis, we used the publicly available so-called SCCS macros (defined as the scripts used to perform the analyses) for SAS.^16,24^ The IRR for IS was calculated as the incidence of IS during the risk period divided by the incidence of IS during the control period. The model included the IS event (present or absent) as the dependent variable, the period (risk or control) as the independent variable, and the following covariates for adjustment: age as a categorical variable (encoded as months since birth date) and RV vaccination – with a 7-day risk period after it – as a binary variable (present or absent).

Based on preliminary queries of the databases, it was estimated that approximately 40 cases of IS with an RV GE claim during the first year of age would be available for inclusion in the study. With 40 cases in the RV GE group, the study had 80% statistical power to demonstrate an association between RV GE and IS if the IRR was ≥6, considering the risk and control periods defined for the primary objective and adjusting for the age effect.

#### Sensitivity analysis

We performed a sensitivity analysis by assessing the risk of IS following fracture, using the same methods, risk periods, and assumptions used for the RV GE group (Figure 2).

#### Post-hoc analyses

Upon the unexpected observation of an increased risk of IS after fracture claims, we conducted post-hoc analyses to explore the consistency and validity of the results. We applied the same SCCS method as for RV GE group except that, in each post-hoc analysis, the following rationale and criteria were applied:

- Post-hoc analysis A: excluding cases where IS and RV GE claims, or IS and fracture claims, were reported on the same date, thus excluding Day 1 from the risk period and resulting in 6- and 20-day risk periods. The aim was to address a potential visit effect,^25^ which would cluster the reporting of IS and RV GE or fracture at the same medical visit (Day 1).
- Post-hoc analysis B: excluding cases where IS and RV GE claims were reported on the same date, and cases with a record of RV vaccination at any time. The aim was to control for a potential visit effect and to ensure that RV vaccination would not reflect an RV GE episode.
- Post-hoc analysis C: excluding cases where IS and RV GE claims, or IS and fracture claims, were reported on the same date, and considering the following risk periods: from Day −6 to Day −1 and from Day −21 to Day −1 (where Day −1 is considered as the day before RV GE or fracture). The aim was to test whether a similar risk of IS was observed during the period before RV GE or fracture, while still controlling for the visit effect.

## Results

### Subject disposition

**Figure 3.**
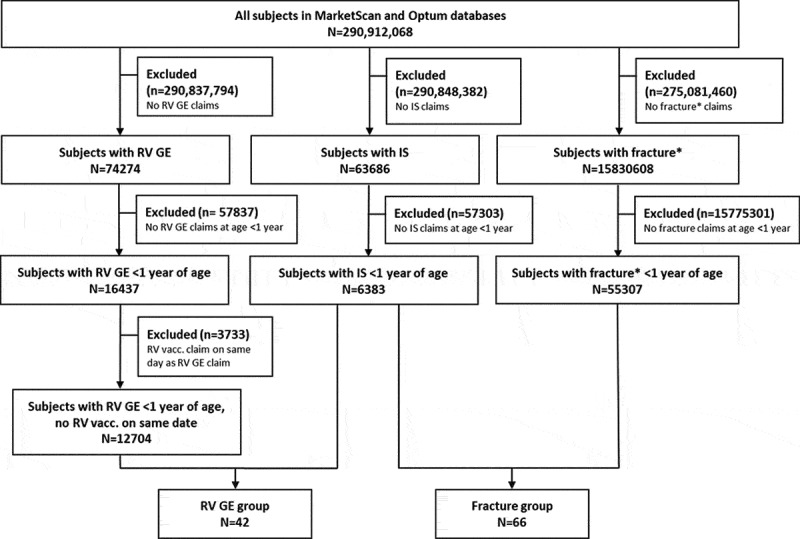
Disposition of subjects in the study. IS, intussusception; N, number of subjects; n, number of subjects excluded (with the reason for exclusion); RV GE, rotavirus gastroenteritis; RV vacc., rotavirus vaccination. * Clavicle fracture and fracture at birth were excluded from this study

A total of 290,912,068 subjects were screened over the period of January 2003–December 2017 in MarketScan and May 2000–December 2017 in Optum. From these, 42 subjects presented claims for RV GE and IS (RV GE group) and 66 presented claims for fracture and IS (fracture group) in the first year of life (Figure 3). There were no instances of both a RV GE code and a fracture code identified for a single subject.

### Descriptive data

**Figure 4.**
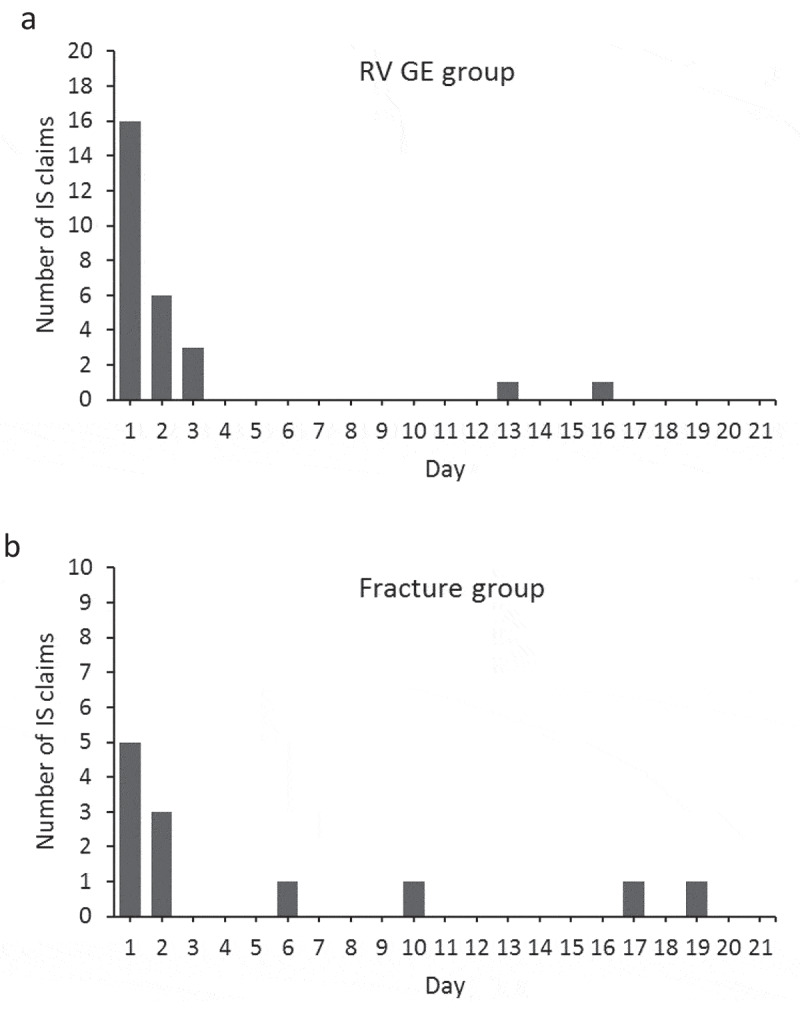
Distribution of intussusception claims during the 21-day risk period after RV GE (a) or fracture (b). IS, intussusception; RV GE, rotavirus gastroenteritis. Day 1 was defined as the day of RV GE or fracture claim

Table 1 shows the demographic and epidemiological characteristics of the subjects. In the RV GE group, 71.4% of subjects had a birth diagnosis recorded. Subjects in the RV GE group presented claims for RV GE at a mean age of 25.2 weeks, and for IS at 26.7 weeks. While 9 (20.9%) claims of IS occurred before RV GE, 25 (58.1%) claims of IS occurred in the 7-day period after RV GE, and 2 (4.7%) occurred between Days 8 and 21 after RV GE (Table 1). When we analyzed the distribution of IS claims over the Day 1–Day 21 risk period, we found most IS claims recorded on the day of RV GE (i.e. Day 1), followed by Days 2 and 3 (Figure 4).

**Table 1.** Demographic and epidemiological characteristics of study subjects

Characteristic	RV GE group N = 42	Fracture group N = 66
Female gender, n (%)	22 (52.4)	23 (34.8)
Birth diagnosis available, n (%)	30 (71.4)	48 (72.7)
Age in weeks at IS, mean (SD)	26.7 (13.9)	24.4 (12.8)
Age in weeks at RV GE or fracture, mean (SD)	25.2 (14.6)	24.8 (15.0)
IS episodes, n (%)		
Before event	9 (20.9)	33 (47.1)
Day 1–Day 7	25 (58.1)	9 (12.9)
Day 8–Day 21	2 (4.7)	3 (4.3)
After Day 21	7 (16.3)	25 (35.7)
Subject-weeks		
Before event	1054.0	1632.1
Day 1–Day 7	42.0	74.6
Day 8–Day 21	84.0	144.3
After Day 21	857.9	1298.0

IS, intussusception; N, number of subjects in the group; n (%), number (percentage) of subjects in a given category; RV GE, rotavirus gastroenteritis; SD, standard deviation.

Day 1 was defined as the day of RV GE or fracture claim.

Demographic characteristics in the fracture group were similar to those in the RV GE group, except for a slightly higher percentage of males (Table 1). The mean ages at IS and at fracture were similar to those observed in the RV GE group for IS and RV GE; however, most of the IS claims occurred before fracture (n: 33; 47.1% of the cases) or in the period more than 21 days after fracture (n: 25; 35.7%). Considering the Day 1–Day 21 risk period, the day with most IS claims recorded was the day of fracture (i.e. Day 1) (Figure 4).

**Figure 5.**
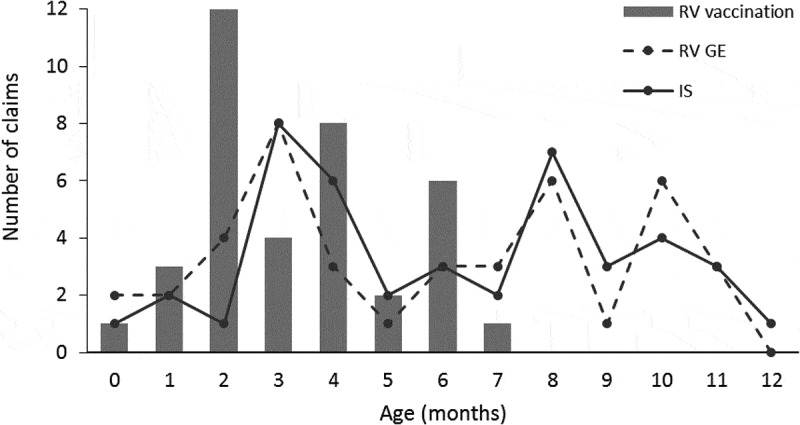
Distribution of claims of rotavirus gastroenteritis, intussusception and rotavirus vaccination by age (RV GE group). IS, intussusception; RV GE, rotavirus; RV GE, rotavirus gastroenteritis

We then analyzed the temporal distribution of RV GE, IS, and RV vaccination claims during the entire first year of life of subjects in the RV GE group. Both the frequencies of RV GE and IS partially overlapped and peaked at months 3, 8, and 10 (Figure 5). We observed three peaks of RV vaccination: at 2, 4, and 6 months, as expected according to the RV vaccination schedule.

In the fracture group, the frequency of fracture was stable across ages, whereas IS peaked at months 3–5 and month 9. The temporal distribution of RV vaccination was similar to that observed in the RV GE group.

### Outcome data, main results, and additional analyses

The IRR of IS after RV GE was 79.6 (95% CI: 38.6–164.4; *p* < .0001) in the 7-day risk period and decreased to 25.5 (13.2–49.2; *p* < .0001) in the 21-day risk period (Table 2). The sensitivity analysis showed an association between IS and fracture for the two risk periods: IRR was 6.1 (3.0–12.7; *p* < .0001) and 2.8 (1.5–5.4; p: 0.0018) in the 7- and 21-day risk periods, respectively (Table 2).

**Table 2.** Incidence rate ratio of intussusception after RV GE or fracture in the main analysis (RV GE group), sensitivity analysis (fracture group), and post-hoc analyses A, B, and C

**Main (RV GE group) and sensitivity (fracture group) analyses**						
	**RV GE group, N= 42**			**Fracture group, N= 66**		
	n	IRR (95% CI)	*p*-value	n	IRR (95% CI)	*p*-value
Risk period						
Day 1–7	41	79.6 (38.6–164.4)	**<0.0001**	67	6.1 (3.0–12.7)	**<0.0001**
Day 1–21	43	25.5 (13.2–49.2)	**<0.0001**	70	2.8 (1.5–5.4)	**0.0018**
**Post-hoc analysis A^a^**						
	**RV GE group, N = 26**			**Fracture group, N = 61**		
	n	IRR (95% CI)	*p*-value	n	IRR (95% CI)	*p*-value
Risk period						
Day 2–7	25	29.9 (12.2–73.3)	**<0.0001**	62	3.1 (1.1–8.7)	**0.0320**
Day 2–21	27	10.7 (4.7–24.3)	**<0.0001**	65	1.7 (0.8–3.8)	0.2049
**Post-hoc analysis B (conducted in RV GE group only)^b^**						
	**RV GE group, N = 18**					
	n	IRR (95% CI)	*p*-value			
Risk period						
Day 2–7	18	21.1 (6.9–64.5)	**<0.0001**			
Day 2–21	18	5.8 (2.0–17.4)	**0.0016**			
**Post-hoc analysis C^c^**						
	**RV GE group, N = 26**			**Fracture group, N = 61**		
	n	IRR (95% CI)	*p*-value	n	IRR (95% CI)	*p*-value
Risk period						
Day −6 – −1	25	4.3 (1.0–19.3)	0.0546	60	0.7 (0.1–5.2)	0.7449
Day −21 – −1	27	2.9 (0.9–8.8)	0.0658	65	1.3 (0.6–3.1)	0.5277

IRR, incidence rate ratio (calculated as the incidence of IS during the risk period divided by the incidence of IS during the control period); IS, intussusception; n, number of cases that presented RV GE and IS or fracture and IS claims in the whole study period (one subject may contribute to several cases if presenting more than one RV GE or fracture claim); RV, rotavirus; RV GE, rotavirus gastroenteritis; 95% CI, 95% Wald confidence interval.

Day 1 was defined as the day of RV GE or fracture claim.

*p*-values in bold indicate statistical significance.

^a^Post-hoc analysis A: analysis excluding cases with IS and RV GE or fracture claims on the same day (Day 1 was excluded from this analysis)

^b^Post-hoc analysis B: analysis excluding cases with IS and RVGE claims on the same day, and cases with RV vaccination (Day 1 was excluded from this analysis)

^c^Post-hoc analysis C: analysis excluding cases with IS and RV GE or fracture claims on the same day and considering risk period before RV GE or fracture (Day −1 is considered as the day before the RV GE claim).

Post-hoc analyses (see details in Methods) are shown in Table 2. In post-hoc analysis A, after excluding Day 1 from the analysis, there was still a statistically significant association between RV GE and IS for both risk periods, but of a lower magnitude (Table 2). In the fracture group, there was still an association for the 6-day period, but no association was detected for the 20-day period (Table 2).

In post-hoc analysis B (in the RV GE group only), after excluding both Day 1 and cases with a recorded RV vaccination, the magnitude of the resulting IRRs was smaller but the association between RV GE and IS remained statistically significant (Table 2).

In post-hoc analysis C, we tested whether we observed the same risk of IS before and after RV GE or fracture. For that purpose, besides excluding again the cases with IS and RV GE or fracture reported on the same date, we defined risk periods before RV GE or fracture. The results showed no statistically significant association between IS and RV GE or fracture when IS occurred before RV GE or before fracture (Table 2).

## Discussion

### Main findings

The aim of this retrospective SCCS was to explore the association between RV GE and the development of IS in infants <1 year of age in the US claims databases.

An increased risk of IS after RV GE was observed in the main analysis. However, the sensitivity analysis also showed an increased risk of IS after fracture – an event for which there is no plausible clinical association with IS – although the magnitude of the risk was lower. Post-hoc analyses conducted to further explore the validity of the data still suggested a possible association between RV GE and IS, but not between fracture and IS.

To our knowledge, this is the first study using claims data to evaluate the association between RV GE and IS. The few previous studies that investigated this association used different designs and data sources.^8,26^ A prospective case–control study showed that experiencing gastroenteritis during the 30 days before an IS diagnosis was associated with IS in children aged 0–59 months (odds ratio: 11.6; 95% CI: 3.2–41.2; *p*-value<0.001).^26^ Although other risk factors for gastroenteritis were not explored in that study, results are consistent with other studies that identified bacterial and viral gastroenteritis as major risk factors for developing IS.^27–31^ While adenovirus is the primary causative agent frequently detected in IS cases, some studies demonstrated an association with rotavirus.^9–11^ In line with these findings, our SCCS method also suggests that a temporal association between RV GE and IS exists.

### Use of claims data

Studies by Restivo and Bines included confirmation of IS cases by medical evaluation and/or laboratory-confirmation of the RV GE diagnosis, and collection of potential risk factor data via questionnaires (e.g. use of antibiotics, history of breastfeeding).^8,26^ Although such methodology is considered more robust than a retrospective database approach, the use of claims databases brings some advantages to our study. Firstly, it limits any recall bias that would be more likely present in studies relying on parents or caretaker interviews. Secondly, it allows to analyze patient-anonymized data from a large population size, which is crucial when studying events with a low background incidence such as IS (34 cases per 100,000 person-years in one study in the US).^32^

There are, however, limitations inherent to the use of claims databases. We cannot exclude a lack of validity and specificity of the IS and RV GE claims, considering that case finding for IS and RV GE was based exclusively on ICD codes declared for the reimbursement of the medical insurance claim. In the pre-vaccination era, RV GE ICD-9 coding was highly specific but showed less than 50% sensitivity.^33^ To our knowledge, there are no published studies establishing the positive predictive value (PPV) of RV GE codes in claims databases in the rotavirus post-vaccination era (i.e. after August 2006 in the US), which is when most of our data were collected. In our study, a correlation between RV GE claims and RV testing claims was not possible, since there are no corresponding laboratory data available in the databases used. In the present study, the use of insurance claims instead of electronic medical records was based on feasibility. During the design of the study, the use of the UK CPRD, a general practitioners’ database which records information on RV testing was explored but then discarded due to the low number of eligible subjects available. On the other hand, regarding IS cases, we relied only on ICD codes. We used specific ICD codes to capture IS, and we excluded nonspecific ICD codes (e.g. “543.9 = Other and unspecified diseases of appendix”). However, this does not inform about diagnosis of certainty. In two previous studies, PPVs of IS diagnosis of 84% and 49% were observed in electronic medical records and claims, respectively.^34,35^ These values reflect the type of data sources, with higher PPVs for validated medical records such as disease registries compared to claims data.

Overall, misclassification of RV GE or IS cannot be excluded in this study. In addition, since the introduction of RV vaccination, changes in laboratory testing practices and, more generally, in clinical practice may bias the reporting of these events in claims databases. Of note, misclassification of RV GE or of IS would generally rather result in an underestimation of the association between the two events.

We observed subjects for whom RV GE and RV vaccination claims were recorded on the same day. Since the labels of the two RV vaccines available in the US recommend caution or delaying vaccination in children presenting with acute gastrointestinal disorders, it is unlikely that both RV GE and RV vaccination truly happened on the same day, suggesting that some of the RV GE claims likely reflect the indication for RV vaccination rather than a true RV GE episode. For this reason, in all analyses, we excluded from the RV GE group any infant with RV GE and RV vaccination claims on the same date, and in post-hoc analysis B, we excluded any subject with an RV vaccination claim. In addition, we observed a distribution of RV vaccination claims consistent with the recommended RV vaccination schedule in the US, which points to the quality of the reporting of these claims.

In our study, IS claims were frequently reported on the same date as RV GE claims. Claims may be clustered on the same date due to a previously described phenomenon known as “Day 0 phenomenon” or “visit effect.”^25^ The visit effect states that collecting information only at specific time points, such as a medical visit, may cause prevalent conditions to be considered as incident, hence affecting the temporal relationship between event and outcome. To account for this effect, we conducted post-hoc analysis A, which excluded Day 1 from the analyses (thus excluding cases with claims of IS and RV GE or fracture on the same day). The reduced IRR observed in this post-hoc analysis compared to the main analysis indeed supports the existence of a visit effect in the distribution of RV GE or fracture and IS claims.

Regarding potential heterogeneity between MarketScan and Optum, we did not observe any discordance between the two databases, since we obtained results consistent between data sources, and consistent with the main analysis results when data were analyzed separately for each database (data not shown).

### Strengths and limitations of the study design

The SCCS method implicitly controls for time-invariant confounders, whether known or unknown. Since age could be a confounding factor for outcomes included in this study, and because RV vaccination is also age-specific, analyses were adjusted for both age and history of RV vaccination. The SCCS has been largely used to assess the association between RV vaccination and IS, and the risk periods used in the present analyses are those described in previous studies and now universally accepted.^13^

We also tested the assumption of a symmetrical pattern of IS reporting by analyzing risk periods before RV GE (post-hoc analysis C). This analysis did not detect an association between IS (occurring first) and RV GE (occurring later), which emphasizes the findings of our main analysis pointing toward a possible association between RV GE and IS in a sequential manner.

### Quality indicator group findings

The main strength of our design is the inclusion of a quality indicator group that considers a medical event, fracture, for which no known nor biologically plausible association with IS exists. The aim of this sensitivity analysis was to provide information about the validity of the claims data, and indeed the results suggest a potential quality issue since a positive temporal association was observed between fracture and IS. The lower—but still significant—association detected in the fracture group through our sensitivity analysis indicates that there is a part of spuriousness in the data, which is difficult to quantify. To partly address this issue, we also excluded cases with claims of IS and fracture on the same date in the fracture group, in order to mitigate a potential visit effect (post-hoc analysis A). When these cases were excluded, the risk of IS after fracture further decreased or disappeared depending on the risk period considered, which could point to a visit effect in the fracture group as well.

The present study is more likely to have investigated medical encounters due to disease symptoms rather than true disease onsets. The use of a broader study endpoint (e.g. all-cause gastroenteritis) as in other studies^7,26^ may have limited potential outcome misclassification and could have increased sensitivity, but would not have answered our research question regarding the role of rotavirus-specific gastroenteritis in the etiology of IS. In general, the interpretation of the results of this study should take into account the nature of claims data, which are a basis for payments and are not necessarily reflecting the actual disease onset.

In conclusion, we observed a temporal positive association between RV GE and IS in US insurance claims databases. However, because of its limitations, this study should be considered as a preliminary investigation. Prospective studies and studies using validated electronic medical records are needed to confirm and quantify more precisely the association between RV GE and IS.

## Data sharing statement

The data that support the findings of this study are available from Truven Health MarketScan Commercial Claims and Encounters and from Optum Clinformatics Data Mart, but restrictions apply to the availability of these data. The data were used under license and so are not publicly available.

## References

[1] Bines JE, Kohl KS, Forster J, Zanardi LR, Davis RL, Hansen J, Murphy TM, Music S, Niu M, Varricchio F, et al. Acute intussusception in infants and children as an adverse event following immunization: case definition and guidelines of data collection, analysis, and presentation. Vaccine. 2004;22(5–6):569–74. doi:10.1016/j.vaccine.2003.09.016. 14741146

[2] Jiang J, Jiang B, Parashar U, Nguyen T, Bines J, Patel MM. Childhood intussusception: a literature review. PLoS One. 2013;8:e68482. doi:10.1371/journal.pone.0068482. 23894308 PMC3718796

[3] Mandeville K, Chien M, Willyerd FA, Mandell G, Hostetler MA, Bulloch B. Intussusception: clinical presentations and imaging characteristics. Pediatr Emerg Care. 2012;28:842–44. doi:10.1097/PEC.0b013e318267a75e. 22929138

[4] Tate JE, Simonsen L, Viboud C, Steiner C, Patel MM, Curns AT, Parashar UD. Trends in intussusception hospitalizations among US infants, 1993–2004: implications for monitoring the safety of the new rotavirus vaccination program. Pediatrics. 2008;121(5):e1125–32. doi:10.1542/peds.2007-1590. 18450856 PMC2680116

[5] Clark AD, Hasso-Agopsowicz M, Kraus MW, Stockdale LK, Sanderson CFB, Parashar UD. Update on the global epidemiology of intussusception: a systematic review of incidence rates, age distributions and case-fatality ratios among children aged <5 years, before the introduction of rotavirus vaccination. Int J Epidemiol. 2019;48(4):1316–1326. doi:10.1093/ije/dyz028. PMC6693807 30879038

[6] Marsicovetere P, Ivatury SJ, White B, Holubar SD. Intestinal intussusception: etiology, diagnosis, and treatment. Clin Colon Rectal Surg. 2017;30:30–39. doi:10.1055/s-0036-1593429. 28144210 PMC5179276

[7] Fotso Kamdem A, Vidal C, Pazart L, Leroux F, Pugin A, Savet C, Sainte-Claire Deville G, Guillemot D, Massol J. A case-control study of risk factors for intussusception among infants in eastern France after the introduction of the rotavirus vaccine. Vaccine. 2019;37(32):4587–93. doi:10.1016/j.vaccine.2019.02.053. 30851968

[8] Bines JE, Liem NT, Justice FA, Son TN, Kirkwood CD, de Campo M, Barnett P, Bishop RF, Robins-Browne R, Carlin JB. Risk factors for intussusception in infants in Vietnam and Australia: adenovirus implicated, but not rotavirus. J Pediatr. 2006;149(4):452–60. doi:10.1016/j.jpeds.2006.04.010. 17011313

[9] Mansour AM, El Koutby M, El Barbary MM, Mohamed W, Shehata S, El Mohammady H, Mostafa M, Riddle MS, Sebeny PJ, Young SYN, et al. Enteric viral infections as potential risk factors for intussusception. The Journal of Infection in Developing Countries. 2013;7(1):28–35. doi:10.3855/jidc.2321. 23324817

[10] Konno T, Suzuki H, Kutsuzawa T, Imai A, Katsushima N, Sakamoto M, Kitaoka S, Tsuboi R, Adachi M. Human rotavirus infection in infants and young children with intussusception. J Med Virol. 1978;2(3):265–69. doi:10.1002/jmv.1890020310. 212529

[11] Minney-Smith CA, Levy A, Hodge M, Jacoby P, Williams SH, Carcione D, Roczo-Farkas S, Kirkwood CD, Smith DW. Intussusception is associated with the detection of adenovirus C, enterovirus B and rotavirus in a rotavirus vaccinated population. J Clin Virol Offl Publ Pan Am Soc Clin Virol. 2014;61(4):579–84. doi:10.1016/j.jcv.2014.10.018. 25464971

[12] Jonesteller CL, Burnett E, Yen C, Tate JE, Parashar UD. Effectiveness of rotavirus vaccination: a systematic review of the first decade of global postlicensure data, 2006–2016. Clinical Infect Dis Offl Publ Infectious Dis Soc Am. 2017;65:840–50. doi:10.1093/cid/cix369. 28444323

[13] Rosillon D, Buyse H, Friedland LR, Ng SP, Velazquez FR, Breuer T. Risk of intussusception after rotavirus vaccination: meta-analysis of postlicensure studies. Pediatr Infect Dis J. 2015;34:763–68. doi:10.1097/INF.0000000000000715. 26069948

[14] Petersen I, Douglas I, Whitaker H. Self controlled case series methods: an alternative to standard epidemiological study designs. BMJ (Clin Res Ed). 2016;354:i4515. 10.1136/bmj.i4515 27618829

[15] Hansen L The Truven Health MarketScan Databases for life sciences researchers. Truven Health Analytics, 2017.

[16] Using SAS for self-controlled case series studies. Easy Internet Solutions LTD, 2018.

[17] Warren-Gash C, Blackburn R, Whitaker H, McMenamin J, Hayward AC. Laboratory-confirmed respiratory infections as triggers for acute myocardial infarction and stroke: a self-controlled case series analysis of national linked datasets from Scotland. Eur Respir J. 2018;51(3):1701794. Published 2018 Mar 29. doi:10.1183/13993003.01794-2017 PMC5898931 29563170

[18] Warren-Gash C, Hayward AC, Hemingway H, Denaxas S, Thomas SL, Timmis AD, Whitaker H, Smeeth L. Influenza infection and risk of acute myocardial infarction in England and Wales: a CALIBER self-controlled case series study. J Infect Dis. 2012;206(11):1652–59. doi:10.1093/infdis/jis597. 23048170 PMC3488196

[19] Thomas SL, Minassian C, Ganesan V, Langan SM, Smeeth L. Chickenpox and risk of stroke: a self-controlled case series analysis. Clinical Infect Dis Offl Publ Infectious Dis Soc Am. 2014;58(1):61–68. doi:10.1093/cid/cit659. PMC3864501 24092802

[20] Farrington CP. Control without separate controls: evaluation of vaccine safety using case-only methods. Vaccine. 2004;22(15–16):2064–70. doi:10.1016/j.vaccine.2004.01.017. 15121324

[21] Weldeselassie YG, Whitaker HJ, Farrington CP. Use of the self-controlled case-series method in vaccine safety studies: review and recommendations for best practice. Epidemiol Infect. 2011;139(12):1805–17. doi:10.1017/S0950268811001531. 21849099

[22] Whitaker HJ, Ghebremichael-Weldeselassie Y, Douglas IJ, Smeeth L, Farrington CP. Investigating the assumptions of the self-controlled case series method. Stat Med. 2018;37(4):643–58. doi:10.1002/sim.7536. 29094391

[23] Optum. Optum clinformatics data mart. Optum, 2014.

[24] Whitaker HJ, Farrington CP, Spiessens B, Musonda P. Tutorial in biostatistics: the self-controlled case series method. Stat Med. 2006;25(10):1768–97. doi:10.1002/sim.2302. 16220518

[25] Jacobsen SJ, Sy LS, Ackerson BK, Chao CR, Slezak JM, Cheetham TC, Takhar HS, Velicer CM, Hansen J, Klein NP. An unmasking phenomenon in an observational post-licensure safety study of adolescent girls and young women. Vaccine. 2012;30(31):4585–87. doi:10.1016/j.vaccine.2012.04.103. 22580356

[26] Restivo V, Costantino C, Giorgianni G, Cuccia M, Tramuto F, Corsello G, Casuccio A, Vitale F. Case–control study on intestinal intussusception: implications for anti-rotavirus vaccination. Expert Rev Vaccines. 2018;17(12):1135–41. doi:10.1080/14760584.2018.1546122. 30407079

[27] Restivo V, Costantino C, Tramuto F, Vitale F. Hospitalization rates for intussusception in children aged 0-59 months from 2009 to 2014 in Italy. Hum Vaccin Immunother. 2017;13:445–49. doi:10.1080/21645515.2017.1264784. 28075671 PMC5328208

[28] Nylund CM, Denson LA, Noel JM. Bacterial enteritis as a risk factor for childhood intussusception: a retrospective cohort study. J Pediatr. 2010;156:761–65. doi:10.1016/j.jpeds.2009.11.026. 20138300

[29] Bhisitkul DM, Todd KM, Listernick R. Adenovirus infection and childhood intussusception. Am J Dis Child. 1960;1992:1331–33. 10.1001/archpedi.1992.02160230089026 1415074

[30] Selvaraj G, Kirkwood C, Bines J, Buttery J. Molecular epidemiology of adenovirus isolates from patients diagnosed with intussusception in Melbourne, Australia. J Clin Microbiol. 2006;44:3371–73. doi:10.1128/JCM.01289-06. 16954276 PMC1594688

[31] Robinson CG, Hernanz-Schulman M, Zhu Y, Griffin MR, Gruber W, Edwards KM. Evaluation of anatomic changes in young children with natural rotavirus infection: is intussusception biologically plausible? J Infect Dis. 2004;189:1382–87. doi:10.1086/382655. 15073674

[32] Tate JE, Yen C, Steiner CA, Cortese MM, Parashar UD. Intussusception rates before and after the introduction of rotavirus vaccine. Pediatrics. 2016;138(3):e20161082. doi:10.1542/peds.2016-1082. PMC11973882 27558938

[33] Hsu VP, Staat MA, Roberts N, Thieman C, Bernstein DI, Bresee J, Glass RI, Parashar UD. Use of active surveillance to validate international classification of diseases code estimates of rotavirus hospitalizations in children. Pediatrics. 2005;115(1):78–82. doi:10.1542/peds.2004-0860. 15629984

[34] Schollin Ask L, Svensson JF, Olen O, Ortqvist A. Clinical presentation of intussusception in Swedish children under 3 years of age and the validity of diagnostic coding. Pediatr Surg Int. 2019;35:373–81. doi:10.1007/s00383-018-4421-3. 30478702 PMC6394471

[35] Hoffman V, Abu-Elyazeed R, Enger C, Esposito DB, Doherty MC, Quinlan SC, Skerry K, Holick CN, Basile P, Friedland LR. Safety study of live, oral human rotavirus vaccine: A cohort study in United States health insurance plans. Hum Vaccin Immunother. 2018;14(7):1782–90. doi:10.1080/21645515.2018.1450123. 29533129 PMC6067866

